# Characterization of patients with clonal mast cells in the bone marrow with clinical significance not otherwise specified

**DOI:** 10.1016/j.eclinm.2024.103043

**Published:** 2025-01-10

**Authors:** Thomas Ballul, Vito Sabato, Peter Valent, Olivier Hermine, Olivier Lortholary, Julien Rossignol, CEREMAST study group, Thomas Ballul, Vito Sabato, Cristina Bulai Livideanu, Antoine Neuraz, Julie Agopian, Fabienne Brenet, Patrice Dubreuil, Didier G. Ebo, Michiel Beyens, Richard Lemal, Olivier Tournilhac, Louis Terriou, David Launay, Laurence Bouillet, Catharina Chatain, Clément Gourguechon, Gandhi Damaj, Stéphane Durupt, Celine Greco, Laurent Frenzel, Christine Bodemer-Skandalis, Laura Polivka, Marine Madrange, Cécile Meni, Hassiba Bouktit, Anne Florence Bellais, Jean-Marc Durand, Marie Gousseff, Edwige Le Mouel, Mohamed Hamidou, Antoine Neel, Dana Ranta, Mathilde Niault, Aurélie Schiffmann, Stéphane Barete, Michel Arock, Danielle Canioni, Thierry Jo Molina, Julie Bruneau, Mélanie Vaes, Violaine Havelange, Hassan Faour, Nicolas Garcelon, Rose-Marie Javier, Fabien Pelletier, Florence Castelain, Denis Vincent, Frédérique Retornaz, Quentin Cabrera, Patricia Zunic, Philippe Guilpain, Marie Pierre Gourin, Ewa Wierzbicka–Hainaut, Jean François Viallard, Christian Lavigne, Cyrille Hoarau, Ludovic Lhermitte, Maël Heiblig, Roland Jaussaud, Peter Valent, Olivier Hermine, Olivier Lortholary, Julien Rossignol

**Affiliations:** a French Reference Center for Mastocytosis (CEREMAST), Paris Cité University, Necker – Enfants Malades University Hospital, APHP, Paris, France; b Department of Immunology Allergology and Rheumatology University of Antwerp and Antwerp University Hospital, Antwerp, Belgium; c Division of Hematology and Hemostaseology, Department of Internal Medicine I, Medical University of Vienna, Austria; d Ludwig Boltzmann Institute for Hematology and Oncology, Medical University of Vienna, Austria

**Keywords:** Mastocytosis, Anaphylaxis, Osteoporosis, *KIT* D816V, Mast cells

## Abstract

**Background:**

Systemic mastocytosis (SM) diagnosis requires the presence of 3 minor criteria or 1 major and 1 minor criterion according to the WHO 2016 classification. The aim of this study was to characterize patients with 1 or 2 minor SM criteria including *KIT 816* mutation and/or aberrant expression of CD2 and/or CD25 on bone marrow (BM) mast cells (MCs), but without MC activation syndrome (MCAS) criteria.

**Methods:**

We included eligible patients from two countries diagnosed between 2011 and 2021. These patients are reported herein as monoclonal MC with clinical significance (MMCS). MMCS patients were compared with 432 patients with indolent SM (ISM) and 51 with BM mastocytosis (BMM) from the CEREMAST database.

**Findings:**

Overall, 51 patients with MMCS were included. MMCS patients with (n = 29) or without (n = 22) *KIT* 816 mutation did not differ significantly with regard to the prevalence of anaphylaxis and basal tryptase level. Anaphylaxis, often in the context of hymenoptera venom allergy, was more frequent in MMCS than in ISM (78% vs 35%, respectively; p < 0.001). Osteoporosis was similarly prevalent in MMCS and BMM (45% vs 32%, p = ns). The median baseline serum tryptase level was lower in MMCS compared with ISM or BMM (13 vs 26 vs 23 ng/mL, respectively; p < 0.001). Hereditary alpha-tryptasemia was similarly represented in MMCS and BMM (14.3% vs 19.7% respectively, p = ns).

**Interpretation:**

Clonal BMMCs may be associated with clinically relevant symptoms even if criteria for SM or MCAS are not fulfilled. These MMCS patients may require specific management and follow-up to capture potential transition to SM and/or MCAS.

**Funding:**

None.

**Research in context**

**Evidence before this study**

Systemic mastocytosis (SM) are defined according to the WHO and ICC classifications and are mostly associated with *KIT* D816V mutations. Among the non-advanced SM, bone marrow mastocytosis (BMM, i.e. without skin involvement) is a form whose prevalence was, until recently, greatly underestimated.

Indeed, recent studies have shown that the prevalence of *KIT* D816V mutation in peripheral blood is more frequent than expected in patients with hymenoptera venum allergy or idiopathic anaphylaxis with normal tryptase level. These data have led to an increase in the screening (and therefore detection) of *KIT* D816V mutation and/or atypical mast cells expressing CD2/CD25 in bone marrow. In these patients, BMM is diagnosed when there is one additional major criterion or 2 other minor WHO criteria. However, we are increasingly seeing patients without cutaneous mastocytosis and with only 1–2 minor WHO criteria in the bone marrow (including *KIT* mutation and/or aberrant expression of CD2/CD25). In the absence of the 3 criteria for mast cell activation (MCA) syndrome, this condition had not been classified and no study involving large numbers of patients have yet been published. We propose to name this condition “monoclonal mast cells with clinical significance” (MMCS).

**Added value of this study**

This study shows that MMCS is a predisposing condition for allergy (especially for hymenoptera venom) and symptoms of MCA, independently of the presence of *KIT* mutation. MMCS patients have lower number of MCA symptoms compared with indolent SM and BMM. In addition, MMCS is frequently associated with osteoporosis as observed in BMM but with lower basal serum tryptase level. Thus, the diagnosis of MMCS is more challenging than BMM.

**Implications of all the available evidence**

MMCS is a clonal hematological disorder, most certainly underdiagnosed and associated with debilitating and/or life-threatening complications. MMCS diagnosis involves specialized examinations, including ddPCR and flow cytometry, integrated into the SM diagnostic workup. This study may greatly help to improve its detection and may also give physicians new insights on mast cell disorders diagnosis. The diagnosis of MMCS requires the physician to implement specific preventive measures (as in mastocytosis), including increased duration of desensitization to hymenoptera venom, precautionary measures for anesthetic risk, and osteoporosis prevention. Finally, it may also help in the future to design therapeutic protocols in which this entity will be included.

## Introduction

Mastocytosis is characterized by accumulation of mast cells (MCs) in various tissues. WHO 2022 classified mastocytosis in different subgroups including cutaneous mastocytosis (CM), mast cell sarcoma and systemic mastocytosis (SM). Indolent SM (ISM) is defined by either the association of the major criterion (i.e. presence of multifocal clusters of abnormal MC) with a minor criterion or by 3 minor criteria without any C-finding and no more than 1B-finding, in the absence of associated hematological neoplasm. ISM without skin lesions, B-finding and basal serum tryptase (BST) < 125 μg/L has been referred as bone marrow mastocytosis (BMM).^1^, ^2^, ^3^, ^4^

Mast cell activation disorders (MCADs) is defined by MC activation (MCA) symptoms and various predisposing conditions including diseases caused by abnormal MCs accumulation as observed in mastocytosis.^5^ Regardless of the severity of the disease, degranulation-related symptoms can dramatically impair the patient's quality of life, cause disability and may even be life-threatening.^6^^,^^7^ The presence of a *KIT* mutation (D816V, in most adults patients) and/or a phenotypically abnormal MCs population are the hallmarks of primary MCAS, mastocytosis and its subcategories according to WHO 2022 classification.^1^^,^^8^

Patients lacking cutaneous mastocytosis and who meet only 1 or 2 minor WHO criteria are not classified as SM^1^ and clinical features they potentially present (including allergy, osteoporosis and symptoms of MCA) have not been extensively described yet.^9^, ^10^, ^11^ They are classified as primary or clonal MCAS if they fulfill all criteria of MCAS (typical symptoms, +20% and +2 ng/mL tryptase elevation during MCA episode, and response to MC stabilizing agent).^5^ If criteria of MCAS are not present, these patients have been referred to by the consensus group as monoclonal MCs of unknown significance (MMUS) when no relevant symptoms are present^3^ or as MCAD when less severe symptoms or symptoms of local MC activation are recorded.^3^ By contrast it seems not appropriate to label these patients as having “sub-diagnostic SM”^12^ or “pre-diagnostic indolent SM”,^13^ given that no progression toward SM have been reported.

So far, it remains unknown whether monoclonal MCs in the BM indeed play a role in the symptomatology of mild or local symptoms of MC activation that do not fulfill the MCAS criteria. However, as these symptoms are similar to that we found in patients with BMM or ISM, we hypothesize that this association is of clinical significance. Based on this assumption, we propose to term this condition “monoclonal MCs with clinical significance (MMCS)”. These patients encompass cases with a *KIT codon 816* mutation as well as cases without a *KIT codon 816* mutation but with abnormal CD2 and/or CD25 expression on MCs which counts as an indirect sign of MC clonality.

The diagnosis of MMCS is challenging, given the very limited MC infiltration of the bone marrow, which can go unnoticed for a long time. Several case reports of idiopathic anaphylaxis or venom allergy in patients with MMCS have been reported^9^, ^10^, ^11^ and only one case series has been published.^12^ The small size of these reported cohorts, however, did not allow comparisons between patients with and without *KIT 816* mutation. Thus, the prevalence of allergy and subtype of MCAD among these patients remain unknown. In addition, the prevalence of hereditary alpha tryptasemia (HαT), which has been recently described as an increased copy number of *TPSAB1* gene and characterized by an increased risk of severe anaphylaxis in patients with SM^14^^,^^15^ have not been studied in this specific population.

ISM and especially BMM, are associated with severe anaphylaxis.^11^^,^^16^ These life-threatening MC degranulation events require an extensive diagnostic workup regarding the mandatory implementation of preventive measures. In addition to its involvement in anaphylaxis, ISM is known to be associated with osteoporosis (8%–41%). However, data on the prevalence of this complication in MMCS have not been previously published.^17^^,^^18^

The aim of this study was to (i) characterize specific features, including allergy, symptoms of MCA, and osteoporosis, in patients with MMCS according to *KIT* mutation, to (ii) compare patients with MMCS and those with ISM or BMM, and to (iii) study and compare the prevalence of HαT in these patient populations.

## Methods

### Patients

**Fig. 1:**
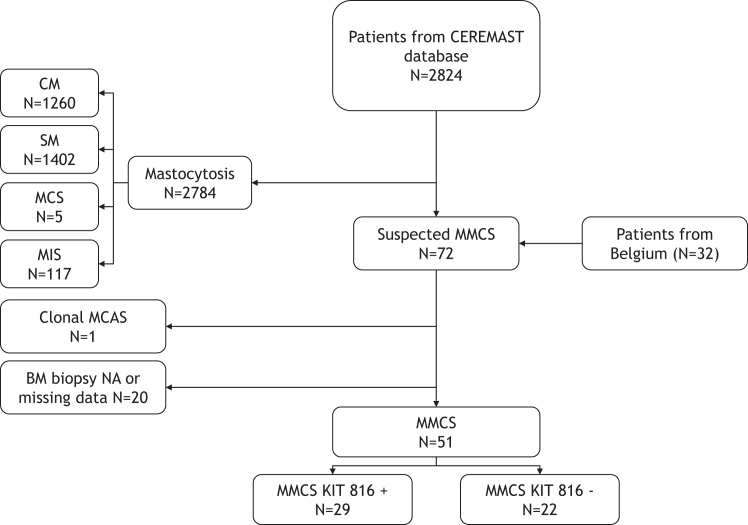
**Flow chart of the study population**. MMCS, monoclonal MC with clinical significance; SM, systemic mastocytosis; CM, cutaneous mastocytosis; MIS, mastocytosis in the skin; MCS, mast cell sarcoma; NA, not available.

We retrospectively reviewed patients with *KIT* codon *816* point mutations or in whom MCs in the BM expressed CD2 and/or CD25 but without fulfilling the criteria of SM according to the WHO 2016 classification.^1^ Patients fulfilling clonal MCAS criteria^5^ were excluded from the study (Fig. 1). Patients were included prospectively in the CEREMAST national database by the French national reference center for mastocytosis (Paris, France) or had been diagnosed at Antwerp University Hospital (Antwerp, Belgium) between 2011 and 2021. Data on the control cohort of 432 patients with ISM were extracted from the CEREMAST database. ISM and BMM were chosen as control groups as they share diagnostic criteria with MMCS.

Diagnosis and classification of mastocytosis were performed according to WHO 2016 classification.^1^ SM was defined as fulfilling the major criterion (i.e. multifocal, dense infiltrates of MCs within the BM biopsy or other extracutaneous organ) and at least one minor criterion (more than 25% of morphologically abnormal MCs in infiltrates; detection of a *KIT 816* point mutation or in other critical regions of *KIT* within the BM or other extracutaneous organ; MCs expressing CD2 and/or CD25, a BST level >20 ng/mL) or fulfilling at least three minor criteria.

MMCS was defined by the presence of an abnormal MC population, characterized by a *KIT 816* mutation in a BM aspirate (detected in a reverse transcription polymerase chain reaction ((RT-PCR, protocol is reported in Supplemental Methods 1) or by droplet digital PCR) and/or an aberrant MC phenotype using immunohistochemistry or flow cytometry (abnormal CD2/CD25 expression) and patients with a *KIT 816* mutation for which MC phenotype data were missing without fulfilling criteria to diagnose SM or MCAS. MMCS were further divided into cases with or without a *KIT 816* mutation. All patients with MMCS presented with symptoms consistent with some MC activation and/or a known IgE-dependent allergy, but did not fulfilled criteria of MCAS (typical symptoms, +20% and +2 ng/mL tryptase elevation during MCA episode, and response to MC stabilizing agent).^5^ Supplemental Tables S1 and S2 summarize inclusion criteria. Data collection methods are reported in Supplemental Methods 2.

### Ethical statement

All the patients in France were enrolled in a prospective study sponsored by AFIRMM. The AFIRMM study was approved by the local investigational review board (Comité de Protection des Personnes Ile-de-France, Pitié-Salpêtrière, France; reference: 93–00). All patients from Belgium were diagnosed at Antwerp University Hospital (Antwerp, Belgium), and approval for this study was obtained from the local ethical committee (reference number B300201316408). This study was carried out in compliance with the principles of the Declaration of Helsinki. Written informed consent was obtained from the patients for the publication of data included in this article.

### Statistical analysis

Categorical variables were analyzed as the frequency (percentage), and continuous variables were analyzed as the median [interquartile range (IQR)]. Groups were compared using Fisher's test or a chi-squared test (for categorical variables) and the Wilcoxon-Mann-Whitney test (for continuous variables). Non-parametric tests are employed in this study due to the violation of normality assumptions in the data, making them more suitable for analyzing ordinal data or distributions with outliers. Normality assumptions was assessed by visual inspection using histograms and boxplots. We performed multivariate analyses using logistic regression, comparing MMCS vs BMM. We kept all the variables with p-value ≤0.2 along with age and sex. All statistical analyses were performed using R software (version 4.0.3, R Foundation for Statistical Computing, Vienna, Austria: www.R-project.org).

### Role of the funding source

This study was conducted by the CEREMAST study group and did not received any external funding source.

## Results

### General characteristics of the study participants

In an initial screening step, 2824 patients registered in the CEREMAST database with proven atypical (on pathological examination or BM smear) and/or *KIT* mutated and/or phenotypically abnormal MCs were identified. Among these patients, 40 were found to be potentially eligible for MMCS criteria. One center in Belgium contributed with data on 32 potentially eligible patients. After all the medical files had been reviewed, 51 patients were found to meet the diagnostic criteria for MMCS (including a mandatory BM biopsy) (Fig. 1). Of these 51 patients, 29 met the criteria for “MMCS KIT 816+” and 22 met the criteria for “MMCS KIT 816−”.

The general characteristics of the MMCS, ISM and BMM group at diagnosis are summarized in Table 1. The median [IQR] age was significantly greater in the MMCS group than in the ISM group (59 [46–64] vs 46 [33–56]; p < 0.001). There was a non-significant trend toward a lower proportion of women in the MMCS group compared to ISM group (52% vs 65%, p = 0.073). The median time from symptom onset to diagnosis was shorter in the MMCS group than in the ISM group (2 [0–6] years vs 5 [1–10] years; p < 0.001). Such differences were not observed comparing MMCS to BMM (Table 1). The groups did not differ significantly in terms of previous or ongoing neoplastic, cardiovascular and autoimmune comorbidities. Supplemental Table S3 shows patient characteristics by country.

**Table 1:** Characteristics of patients with MMCS and patients with ISM or BMM (controls).

General characteristics	MMCS N = 51	ISM N = 432	p-value	BMM N = 51	p-value
Age at diagnosis, y, median [IQR]	59 [46–64]	46 [33–56]	<0.001	54 [45–62]	ns
Time between symptom onset and diagnosis, y, median [IQR]	2 [0–6]	5 [1–10]	<0.001	2.0 [1.0–6.0]	ns
Sex ratio, M/F	0.96	0.54	ns	1.04	ns
**Medical history**	**n/N (%)**	**n/N (%)**	**p-value**	**n/N (%)**	**p-value**

Malignant tumor	5/49 (10)	42/382 (11)	ns	4/48 (8)	ns
Cardiovascular disease	6/45 (13)	61/360 (17)	ns	10/48 (21)	ns
Autoimmune disease	1/50 (2)	26/375 (7)	ns	5/47 (11)	ns
**Diagnostic criteria**					
Aberrant CD2 expression	23/49 (47)	195/216 (90)	<0.001	46/48 (96)	<0.001
Aberrant CD25 expression	33/49 (67)	207/218 (95)	<0.001	48/49 (98)	<0.001
*KIT* mutation status			<0.001		<0.001
Not screened	0/51	3/281		2/51	
D816V	29/51 (57)	240/281 (86)		45/51 (92)	
Other *KIT* mutations	0/51 (0)	4/281 (1)		2/51 (4)	
Wild-type *KIT*	22/51 (43)	34/281 (12)		2/51 (4)	
Baseline serum tryptase level >20 ng/mL	10/48 (21)	235/364 (65)	<0.001	32 (65)	<0.001

### Diagnostic criteria

As expected, patients with ISM met one to four minor diagnostic criteria from the WHO 2016 classification.^1^ Aberrant MC surface expression of CD2 and CD25 was respectively observed in 43% and 63% of the patients with MMCS, vs 90% and 95% of the patients with ISM (p < 0.001). *KIT 816* mutations were more frequent in the ISM group (88%, vs 57% in the MMCS group; p < 0.001). The proportion of patients with a BST >20 ng/mL was much lower in the MMCS group than in the ISM group (21% vs 65%, respectively; p < 0.001).

### Clinical presentations

Overall, 23 patients presented with allergy investigated by both allergy skin and specific IgE tests (including Hymenoptera venom allergy (HVA) N = 20 and drug allergy N = 3) and among them, 6 presented with mild symptoms of MCA. Twenty-three patients had only symptoms of MCA and 5 patients presented with idiopathic anaphylaxis. As expected, none of the patients had the 3 MCAS criteria, and patients with MCA met the criteria for “other MCAD” according to the MCAD classification. Taken together, more than half of our patients (55%) were referred to a physician for allergy or idiopathic anaphylaxis without other symptoms of MCA (Supplemental Figure S1).

The mean (range) number of clinical signs was lower in the MMCS group than in the ISM group (1.9 (0–6) vs 6.3 (4–8), respectively; p < 0.001). Specifically, patients with MMCS were significantly less likely to present with skin involvement including urticaria or pruritus (34%, vs 91% in the ISM group; p < 0.001), gastrointestinal symptoms (43% vs 69%, respectively; p < 0.001), neuropsychiatric symptoms (26% vs 66%, respectively; p < 0.001), musculoskeletal symptoms (15% vs 79%, respectively; p < 0.001), flushing (25% vs 68%, respectively; p < 0.001), and malaise (9.8% vs 41%, respectively; p < 0.001). As for the comparison between MMCS and BMM, skin and gastrointestinal involvements were equally present in both groups (34% vs 27% and 43 vs 41% respectively; p = ns) but MMCS patients had less neuropsychiatric symptoms (26% vs 51%, respectively; p = 0.010), musculoskeletal symptoms (15% vs 47%, respectively; p < 0.001), flushing (25% vs 65%, respectively; p < 0.001) and malaise (9.8% vs 40%, respectively; p < 0.001).

In contrast, anaphylaxis (including its severe manifestations, such as anaphylactic shock and severe angioedema) was more prevalent in the MMCS group (78% vs 35% in the ISM group; p < 0.001). This difference was not observed between the MMCS and the BMM groups (78% vs 76%, respectively).

The prevalence of osteoporosis was higher (albeit not significantly) in the MMCS group (45%) than in the ISM group (31%; p = ns). The same trend was observed between the MMCS and the BMM groups (45% vs 32%, respectively; p = ns). These results are summarized in Table 2.

**Table 2:** Clinical presentation of patients with MMCS and patients with ISM or BMM (controls).

Symptoms	MMCS N = 51	ISM N = 432	p-value	*BMM N = 51*	p-value
	n/N (%)	n/N (%)		n/N (%)	
Anaphylaxis	40/51 (78)	131/376 (35)	*<0.001*	39/51 (76)	ns
Cutaneous (pruritus, excessive sweating, dermographism, urticaria)	17/50 (34)	384/422 (91)	*<0.001*	14 (27)	ns
Gastrointestinal	22/51 (43)	281/410 (69)	*<0.001*	21 (41)	ns
Flushing	13/51 (25)	277/407 (68)	*<0.001*	33 (65)	<0.001
Neuropsychiatric	12/47 (26)	269/405 (66)	*<0.001*	26 (51)	0.010
Musculoskeletal	7/46 (15)	195/248 (79)	*<0.001*	24 (47)	<0.001
Malaises	5/51 (10)	94/228 (41)	*<0.001*	20 (40)	<0.001
Asthenia	8/45 (18)	205/256 (80)	*<0.001*	4 (8)	ns
Splenomegaly	1/42 (2)	19/243 (8)	*ns*	2 (4)	ns
Osteoporosis	10/22 (45)	35/112 (31)	*ns*	8 (32)	ns

To further characterize bone mineral density (BMD) abnormalities, we compared BMD parameters among patients with a T-score <1 (Supplemental Table S4). The median [IQR] femoral T-score was −1.45 [−2.18 to −1.28] in the MMCS group and −1.60 [−2.10 to −1.0] in the ISM group. The median [IQR] lumbar T-score was −2.55 [−2.90; −2.08] in the MMCS group and −2.00 [−2.65 to −1.40] in the ISM group. These intergroup differences were not statistically significant (p = 0.68 for the femur and p = 0.16 for the lumbar region). Supplemental Tables S5 and S6 show adjusted analysis on age, sex, delay from symptoms onset to diagnosis for patient characteristics and clinical presentation, comparing MMCS to ISM and MMCS to BMM, respectively. We found no other significant difference regarding adjusted analysis. Multivariates analysis are shown in Supplemental Table S7A and B (MMCS vs ISM) and 8A-8B (MMCS vs BMM). These highlighted that MMCS diagnosis was significatively shorter when comparing MMCS to ISM, that musculoskeletal involvement, malaises and asthenia were less frequent in the MMCS group compared to ISM, and that malaises, musculoskeletal involvement, and flushing were less frequent when comparing MMCS to BMM. We also noted that multivariate analysis showed that aberrant CD2 expression and *KIT* D816V mutation were more frequent in BMM and ISM compared to MMCS. It was not possible to add the country in this comparison given that only MMCS patients from Belgium were present, without BMM patients.

### Laboratory parameters

The median [IQR] BST level was significantly lower in the MMCS group than both in the ISM group (13 ng/mL [7–17] vs 26 ng/mL [14–51], respectively; p < 0.001) and in the BMM group (13 ng/mL [7–17] vs 23 ng/mL [16–36], respectively; p < 0.001) (Table 3). There were no significant intergroup differences in the other laboratory parameters, including blood cell counts, the proportion of patients with elevated alkaline phosphatase levels, and the total serum IgE level (Table 3). Finally, HαT was found in 4/26 patients in the MMCS KIT 816+ group and in 2/16 patients in the MMCS KIT 816− group (15% and 13%, respectively; p = ns).

**Table 3:** Laboratory characteristics of patients with MMCS and patients with ISM or BMM (controls).

Laboratory characteristic	MMCS N = 51	ISM N = 432	p-value	BMM N = 51	p-value
	Median [IQR]	Median [IQR]		Median [IQR]	
Baseline serum tryptase (ng/mL)	13 [7–17]	26 [14–51]	<0.001	23 [16–36]	<0.001
Hemoglobin (g/dL)	13.9 [13.6–14.8]	13.7 [13–14.6]	ns	13.90 [13.35–14.70]	ns
Platelet count (giga/L)	249 [216–314]	253 [214–293]	ns	246 [219–268]	ns
Leukocyte count (giga/L)	5.91 [5.14–7.16]	6.40 [5.40–7.80]	ns	6.50 [5.60–7.15]	ns
Eosinophil count (giga/L)	0.2 [0.1–0.3]	0.2 [0.1–0.3]	ns	0.2 [0.1–0.2]	ns
IgE (kIU/L)	50 [20–110]	30 [10–70]	ns	40 [20–100]	ns
% of patients with elevated alkaline phosphatase levels	2.4%	4.9%	ns	2.6%	ns

### Treatments received

The proportion of patients treated with H1 antihistamines at diagnosis was lower in the MMCS group than in the ISM group (51% vs 78%, respectively; p < 0.001). H2 antihistamines and montelukast were respectively administered to 36% and 24% of the patients with MMCS and 49% and 29% of the patients with ISM. These intergroup differences were not statistically significant. None of the patients with MMCS had received rapamycin (0%, vs 1.4% in the ISM group; p = ns), interferon alpha-2a (pegylated or not) (0% vs 9%, respectively; p = 0.024) or imatinib (0% vs 4.4%, p = ns). There was no significant intergroup difference in treatment with omalizumab (10% in the MMCS group vs 5.1% in the ISM group; p = ns). An epinephrine self-injection kit was prescribed to all patients with anaphylaxis. Overall, symptomatic medication use was less intense in the MMCS group than in the ISM group (Supplemental Table S9).

Yet, there was no significant difference between MMCS and BMM groups regarding medication, though H1 antihistamines tended to be used more frequently in the BMM group than in the MMCS group (67% vs 51%, respectively; p = 0.089). H2 antihistamines and montelukast were both given to 25% of the patients with BMM. One BMM patient received rapamycin, one received interferon alpha-2a and eight (17%) received Omalizumab (Supplemental Table S9).

### Comparison of MMCS KIT 816+ and MMCS KIT 816−

There was no association between most of symptoms, including anaphylaxis, and the presence of a *KIT 816* mutation. Indeed, the clinical profile of patients with MMCS KIT 816+ was almost similar to that of patients with MMCS KIT 816− (Table 4) with respect to anaphylaxis (in 83% vs 73% of the patients, respectively; p = ns), skin symptoms (24% vs 48%, respectively; p = ns), neuropsychiatric symptoms (21% vs 32%, respectively; p = ns), musculoskeletal involvement (7.1% vs 28%, respectively; p = ns), malaise (3.4% vs 18%, respectively; p = ns), and asthenia (14% vs 24%, respectively; p = ns). However, the MMCS KIT 816+ and MMCS KIT 816- groups were different regarding gastrointestinal symptoms (31% vs 59%, respectively; p = 0.045) and flushes (14% vs 41%, respectively; p = 0.028).

**Table 4:** Clinical presentation and laboratory characteristics of patients with MMCS KIT 816+ vs MMCS KIT 816−.

Characteristic	MMCS KIT 816+ N = 29	MMCS KIT 816- N = 22	p-value
	n/N (%)	n/N (%)	
Anaphylaxis	24/29 (83)	16/22 (73)	ns
Cutaneous (pruritus, excessive sweating, dermographism, urticaria)	7/29 (24)	10/21 (48)	ns
Gastrointestinal	9/29 (31)	13/22 (59)	0.045
Flushes	4/29 (14)	9/22 (41)	0.028
Neuropsychiatric	6/28 (21)	6/19 (32)	ns
Musculoskeletal	2/28 (7.1)	5/18 (28)	ns
Malaise	1/29 (3.4)	4/22 (18)	ns
Asthenia	4/28 (14)	4/17 (24)	ns
Baseline serum tryptase level (ng/mL)	12 (8–16)	13 (6–23)	ns
Hereditary alpha tryptasemia	4/26 (15)	2/16 (12)	ns

### Follow-up of patients with MMCS

Over a median (range) follow-up period of 4.7 years (3–11), we did not observe any appearance of additional MCAS criteria, mastocytosis skin lesions, transformation to advanced SM or death. However, in the absence of significant changes in follow-up parameters (as judged by the attending physicians), none of the patients underwent an additional BM biopsy, therefore none of them was diagnosed with BMM during follow up.

## Discussion

Mastocytosis encompasses a spectrum of MC diseases including cutaneous mastocytosis, systemic mastocytosis, and MC sarcoma defined in the WHO 2016 classification.^1^ However, patients lacking cutaneous mastocytosis and SM criteria because they meet only 1 or 2 minor criteria according to WHO 2016 criteria (including or not the presence of the *KIT 816* mutation) have not previously been well characterized (excluding patients meeting the 3 MCAS criteria so called clonal MCAS). Therefore, the objectives of the present study were to characterize these MMCS patients and to compare them with ISM and BMM patients regarding clinical presentation, including allergy (especially HVA), symptoms of MCA and osteoporosis.

We collected data on 51 patients with genetically and/or phenotypically abnormal MC in BM that did not meet the WHO 2016 criteria for mastocytosis^1^ and MCAS.^5^ Hence, we decided to group herein all the patients under the term “MMCS KIT 816−/+” because of the overlap with systemic mastocytosis and its complications (severe anaphylaxis, osteoporosis)–even though the disease could be considered to be less advanced.

An analysis of the group's characteristics showed that the clinical profile for MMCS differs markedly from patients with ISM. We hypothesize that even though the patients with MMCS had a lower overall symptom burden, the acute, severe nature of their symptoms resulted in a shorter diagnostic delay. Anaphylactic events (i.e. laryngeal angioedema with respiratory distress or even shock) were more frequent in patients with MMCS, whereas other signs of MC activation were more prominent in controls. This striking difference prompts us to draw a distinction between MMCS and both ISM and idiopathic MCAS, whose clinical presentation seems similar to that of ISM.^19^^,^^20^

In addition, the prevalence of MMCS in patients presenting only with osteoporosis remains to be investigated. Indeed, we did not find any significant difference between the MMCS and ISM groups with regard to the prevalence of densitometry-proven osteoporosis. This finding is in line with a recent report^18^ of an association between SM and osteoporosis in 8%–41% of patients that is contrasting to idiopathic MCAS or CM in which osteoporosis is rarely observed.^21^ The clinical presentation of MMCS appeared to be close to BMM. Similarly, Zanotti et al. observed the same differences as we did between BMM and ISM in the present work: a greater median age at diagnosis, a shorter time to diagnosis, more frequent anaphylaxis, and less frequent signs of MC degranulation.^22^ It is also noteworthy that over a median follow-up period of 4.7 years, we did not observe the appearance of any cutaneous lesion or any transformation of MMCS to SM. However, it is possible that some patients developed a BMM but none of the MMCS patients reported in our study have undergone an additional BM biopsy because no clinical and biological worsening were observed that would have justified it. Overall, these data prompt us to hypothesize that MMCS is likely a form of BMM with low MCs burden.

The present study corresponds to the largest yet reported cohort of patients with MMCS. However, their predominantly “anaphylactic” clinical presentation suggests that MMCS is underdiagnosed–especially among patients with so-called idiopathic anaphylaxis.^23^ This hypothesis is supported by recent data from other research groups.^11^^,^^16^ Carter et al. reported that 14% of patients presenting with idiopathic anaphylaxis might have a clonal MC disease.^11^ Similar results were reported in a study in which 5% of the patients referred for severe anaphylaxis were found to have a clonal MC disease, with a *KIT 816* mutation but a normal BST level.^16^ If a certain proportion of patients with idiopathic anaphylaxis does indeed have MMCS, this disease may constitute a real public health issue.^24^ Our present results also suggest that BST as a biomarker is not always reliable; the median [IQR] BST level in our patients with MMCS was 13 ng/mL [7–17] (Table 1), and only 10 of the 48 patients tested (21%) had a BST level above the threshold of 20 ng/mL.

HαT is known to be present in about 5–6% of the general population.^25^ In addition, it is also well known that HαT is more prevalent among patients suffering from mastocytosis (approximately 17%) and that these patients are more prompt to develop severe anaphylaxis.^26^ These data suggest that HαT might somehow promote anaphylaxis, leading to an easier and earlier diagnosis of mastocytosis. In our study, HαT was observed in 14% of our MMCS patients, regardless of *KIT 816* mutation. Despite the high prevalence of anaphylaxis in our MMCS patients, the prevalence of HαT does not appear to be higher than in SM, suggesting that HαT does not explain by itself the prevalence of anaphylaxis in our study.^26^

Our present results were probably influenced by other sources of biases. Firstly, a clinical observation of anaphylaxis and osteoporosis should prompt the physician to screen for SM in a certain number of cases, which would increase the prevalence of these signs in the clinical picture of MMCS. It is likely that some patients with MMCS have few or no symptoms and would not be prescribed a BM biopsy. Secondly, the true prevalence of MMCS in France and Belgium was probably underestimated because we observed great disparity in inclusion between centers. Indeed, there was a marked difference between the patient recruitments in Belgium (with one center alone which is also a center for anaphylaxis, providing 23 patients) and in France (28 patients recruited by a nationwide network). When analyzing patients based on the country of care (Supplemental Table S3), we observed significant differences regarding the age of first symptoms (60 years old [50–64] in Belgium vs 45 years old [40–55] in France, p = 0.004), the diagnostic delay (0 years [0–2] in Belgium vs 4 years [1–9] in France, p = 0.005), and the clinical presentation (cutaneous, gastrointestinal, neuropsychiatric involvements, flushing and asthenia were more frequent in the France group than in Belgium group: 57% vs 4.5%, p < 0.001; 57% vs 26%, p = 0.026; 46% vs 4.3%, p = 0.001; 43% vs 4.3%, p = 0.002; 32% vs 4.3%, p = 0.022, respectively). Thus, Belgian cohort is more representative of MMCS cases diagnosed in a highly specialized allergy center (i.e. referred for anaphylaxis), while the MMCS cases in France are more representative of patients diagnosed with signs of MCA, whether or not associated with anaphylaxis.

These findings, along with the center effect observed due to a high number of diagnoses made at a single center in Belgium, suggest an underdiagnosis of MMCS in other centers outside of Antwerp. This may be related to the less frequent investigation for mastocytosis in patients with anaphylaxis in France, as well as the less frequent use of BM biopsies in that country. Indeed, we identified more than fifty patients in France who potentially met the criteria for MMCS but had not undergone a BM biopsy, necessitating their exclusion from the study. This observation highlights the critical need to establish specific patient pathways for investigating mastocytosis. Taken as a whole, our results suggest that MMCS is a likely under-diagnosed condition.

As recently described, we cannot rule out a possible lack of sensitivity of the investigations performed in our study.^27^ This issue may involve particularly *KIT* mutation assays, immunophenotyping and tryptase levels. Thus, repeating the investigations, or using a more sensitive strategy such as BM MC sorting, might lead some of our patients to be finally diagnosed with BMM. In addition, none of our patients fulfilled all MCAS criteria (Supplementary Table S1). Longer follow-up would probably enable us to obtain full MCAS criteria in some of them (which would lead to the diagnosis of clonal MCAS). Indeed, the increased tryptase level criterion is sometimes difficult to gather in daily practice. As a result, much data regarding these criteria is missing in our cohort, as shown in Supplemental Table S2. Thus, the MMCS entity can serve as a phenotypic description until a potential final diagnosis is made.

The diagnosis of monoclonal MCAS has previously been proposed for a subset of patients with one or two minor criteria without fulfilling the criteria for SM. Akin et al.^10^ reported five patients with idiopathic anaphylaxis, while Sonneck et al.^9^ reported one patient with HVA. In these studies, MCAS criteria were not specified. More recently, Gülen et al.^28^ provided insights on the international classification of MCAS. Clinically, the diagnosis of MCAS requires acute symptoms (whether or not associated with anaphylaxis) involving two of the following four organ systems: cutaneous, gastrointestinal, respiratory, and cardiovascular. Additionally, it requires an increase in acute serum tryptase compared to BST (×1.2 + 2 ng/mL). The diagnosis of monoclonal MCAS should be made in the presence of the three MCAS criteria, along with evidence of clonality.

We find these criteria to be more applicable in clinical settings because (i) the criteria for diagnosing MCAS remain the same, regardless of whether clonality is present, (ii) they do not distinguish between patients who have experienced anaphylaxis and those who have not, and (iii) the biological criterion (tryptase) serves as a reliable indicator of MCA. Therefore, we believe that the entity reported here (i.e. MMCS) simplifies the classification of clonal MCADs without SM criteria: in the presence of the three MCAS criteria, MMAS should be diagnosed, and in the absence of at least one MCAS criterion, MMCS should be diagnosed.

Diagnosing MMCS is critically important in patients with idiopathic trabecular osteoporosis and/or early-onset osteoporosis, even when the BST level is normal. This diagnosis is essential for taking preventive measures, especially for anaphylactic shock in high-risk situations (HVA desensitization and general anesthesia). The treatment of patients with MMCS is currently based on symptomatic medications (H1 and H2 antihistamines, sodium cromoglycate, anti-leukotrienes and omalizumab^29^^,^^30^) as part of a comprehensive, multidisciplinary approach. The treatment of MMCS with tyrosine kinase inhibitors (targeting *KIT 816* or not) warrants investigation.

In conclusion, MMCS is a disorder characterized by MC with signs of clonality in BM without fulfilling SM and MMAS criteria. It may result in osteoporosis and life-threatening complications, such as severe anaphylaxis. MMCS requires a specific diagnostic workup to treat and to prevent the development of complications. A careful individual follow-up of patients with MMCS should be proposed, as the long-term history of *KIT*-mutated or WT forms remains to be described and may particularly evolve towards SM.

## Contributors

T.B. and J.R. collected, assembled and analyzed the data and wrote the manuscript.

V.S. and C.B.L helped to collect, assemble and analyze the data.

A.N. performed the statistical analysis.

T.B., J.R., O.H., V.S., A.N., accessed and verified the underlying data.

J.A., F.B., P.D., D.G.E, R.L., O.T., L.T., D.L., L.B., C.C., C.G., G.D., S.D., C.G., L.F., C.B., L.P, M.M., C.M., H.B., A.F.B., J.M.D., M.G., E.L.M, M.H., A.N., D.R., M.N., A.S., S.B., M.A., D.C., T.J.M., J.B., M.V., V.H., H.F., N.G., R.M.J., F.P., V.D., F.R., Q.C., P.Z., P.G., M.P.G., E.W.H., J.F.V., C.L., C.H., L.L., M.H., R.J., and F.C. helped to collect data and revised the manuscript.

J.R., O.H., V.S., P.V., and O.L. designed the study, supervised the overall project, analyzed the data and approved the final version of the manuscript.

## Data sharing statement

The data that support the findings of this study are available from the corresponding author [O.H.] upon reasonable request.

## Declaration of interests

J.R.: Blueprint Medicines, MSD.

V.S.: Blueprint Medicines, Novartis, Cogent, Telios, Termofisher.

## Appendix A Supplementary data

Supplemental_Data

CEREMAST study group

## References

[1] Valent P., Akin C., Metcalfe D.D. (2017). Mastocytosis: 2016 updated WHO classification and novel emerging treatment concepts. Blood.

[2] Arber D.A., Orazi A., Hasserjian R. (2016). The 2016 revision to the World Health Organization classification of myeloid neoplasms and acute leukemia. Blood.

[3] Valent P., Akin C., Escribano L. (2007). Standards and standardization in mastocytosis: consensus statements on diagnostics, treatment recommendations and response criteria. Eur J Clin Invest.

[4] Pardanani A., Lim K.H., Lasho T.L. (2010). WHO subvariants of indolent mastocytosis: clinical details and prognostic evaluation in 159 consecutive adults. Blood.

[5] Valent P., Hartmann K., Bonadonna P. (2022). Global classification of mast cell activation disorders: an ICD-10-CM–adjusted proposal of the ECNM-AIM consortium. J Allergy Clin Immunol Pract.

[6] Theoharides T.C., Valent P., Akin C. (2015). Mast cells, mastocytosis, and related disorders. N Engl J Med.

[7] Hermine O., Lortholary O., Leventhal P.S. (2008). Case-control cohort study of patients' perceptions of disability in mastocytosis. PLoS One.

[8] Escribano L., Diaz-Agustin B., López A. (2004). Immunophenotypic analysis of mast cells in mastocytosis: when and how to do it. Proposals of the Spanish Network on Mastocytosis (REMA). Cytometry B Clin Cytom.

[9] Sonneck K., Florian S., Müllauer L. (2007). Diagnostic and subdiagnostic accumulation of mast cells in the bone marrow of patients with anaphylaxis: monoclonal mast cell activation syndrome. Int Arch Allergy Immunol.

[10] Akin C., Scott L.M., Kocabas C.N. (2007). Demonstration of an aberrant mast-cell population with clonal markers in a subset of patients with “idiopathic” anaphylaxis. Blood.

[11] Carter M.C., Desai A., Komarow H.D. (2018). A distinct biomolecular profile identifies monoclonal mast cell disorders in patients with idiopathic anaphylaxis. J Allergy Clin Immunol.

[12] Pardanani A., Chen D., Abdelrahman R.A. (2013). Clonal mast cell disease not meeting WHO criteria for diagnosis of mastocytosis: clinicopathologic features and comparison with indolent mastocytosis. Leukemia.

[13] Pardanani A. (2019). Systemic mastocytosis in adults: 2019 update on diagnosis, risk stratification and management. Am J Hematol.

[14] Lyons J.J., Yu X., Hughes J.D. (2016). Elevated basal serum tryptase identifies a multisystem disorder associated with increased TPSAB1 copy number. Nat Genet.

[15] Lyons J.J., Chovanec J., O'Connell M.P. (2021). Heritable risk for severe anaphylaxis associated with increased α-tryptase-encoding germline copy number at TPSAB1. J Allergy Clin Immunol.

[16] Dölle-Bierke S., Siebenhaar F., Burmeister T., Worm M. (2019). Detection of KIT D816V mutation in patients with severe anaphylaxis and normal basal tryptase—first data from the Anaphylaxis Registry (NORA). J Allergy Clin Immunol.

[17] Rossini M., Zanotti R., Orsolini G. (2016). Prevalence, pathogenesis, and treatment options for mastocytosis-related osteoporosis. Osteoporos Int.

[18] Orsolini G., Viapiana O., Rossini M., Bonifacio M., Zanotti R. (2018). Bone disease in mastocytosis. Immunol Allergy Clin North Am.

[19] Álvarez-Twose I., González de Olano D., Sánchez-Muñoz L. (2010). Clinical, biological, and molecular characteristics of clonal mast cell disorders presenting with systemic mast cell activation symptoms. J Allergy Clin Immunol.

[20] Afrin L.B., Self S., Menk J., Lazarchick J. (2017). Characterization of mast cell activation syndrome. Am J Med Sci.

[21] Degboé Y., Eischen M., Apoil P.A. (2019). Higher prevalence of vertebral fractures in systemic mastocytosis, but not in cutaneous mastocytosis and idiopathic mast cell activation syndrome. Osteoporos Int.

[22] Zanotti R., Bonadonna P., Bonifacio M. (2011). Isolated bone marrow mastocytosis: an underestimated subvariant of indolent systemic mastocytosis. Haematologica.

[23] Giannetti M.P., Akin C., Castells M. (2020). Idiopathic anaphylaxis: a form of mast cell activation syndrome. J Allergy Clin Immunol Pract.

[24] Webb L.M., Lieberman P. (2006). Anaphylaxis: a review of 601 cases. Ann Allergy Asthma Immunol.

[25] Chollet M.B., Akin C. (2021). Hereditary alpha tryptasemia is not associated with specific clinical phenotypes. J Allergy Clin Immunol.

[26] Greiner G., Sprinzl B., Górska A. (2021). Hereditary α tryptasemia is a valid genetic biomarker for severe mediator-related symptoms in mastocytosis. Blood.

[27] Boggs N.A., Sun X., Lyons J.J. (2023). Challenges in applying diagnostic criteria for systemic mastocytosis. Blood Adv.

[28] Gülen T., Akin C., Bonadonna P. (2021). Selecting the right criteria and proper classification to diagnose mast cell activation syndromes: a critical review. J Allergy Clin Immunol Pract.

[29] Lemal R., Fouquet G., Terriou L. (2019). Omalizumab therapy for mast cell-mediator symptoms in patients with ISM, CM, MMAS, and MCAS. J Allergy Clin Immunol Pract.

[30] Broesby-Olsen S., Vestergaard H., Mortz C.G. (2018). Omalizumab prevents anaphylaxis and improves symptoms in systemic mastocytosis: efficacy and safety observations. Allergy.

