# Supplementary material for: Characterization of patients with clonal mast cells in the bone marrow with clinical significance not otherwise specified
Source: eClinicalMedicine. 2025 Jan 10;80:103043. doi: 10.1016/j.eclinm.2024.103043 (PMC11773267; doi:10.1016/j.eclinm.2024.103043)
Supplement: Supplemental_Data [file mmc1.docx]

Table of contents

[Supplemental Methods 1 2](#_Toc179643932)

[Supplemental Methods 2 4](#_Toc179643933)

[Supplemental Table 1 5](#_Toc179643934)

[Supplemental Table 2 7](#_Toc179643935)

[Supplemental Table 3 9](#_Toc179643936)

[Supplemental Table 4 10](#_Toc179643937)

[Supplemental Table 5 11](#_Toc179643938)

[Supplemental Table 6 12](#_Toc179643939)

[Supplemental Table 7A 13](#_Toc179643940)

[Supplemental Table 7B 13](#_Toc179643941)

[Supplemental Table 8A 14](#_Toc179643942)

[Supplemental Table 8B 14](#_Toc179643943)

[Supplemental Table 9 15](#_Toc179643944)

[Supplemental Figure 1 16](#_Toc179643945)

# Supplemental Methods 1

Total RNA was extracted from cutaneous biopsy or bone marrow aspirate using the RNeasy Mini kit (Qiagen). RNA was reverse transcribed into cDNA using the AffinityScript Multiple Temperature cDNA Synthesis kit (Agilent Technologies), with random primers and oligodT as recommended by the manufacturer. To detect specifically the codon 816 mutation into exon 17, the *c-KIT* coding sequence was amplified in duplicate in two different tubes using the Phire Hot Start DNA Polymerase (Finnzymes) and the primers U2 and L1 listed in Table 1. PCR reaction was performed with 35 cycles at 98°c for 5 seconds, 60°c for 5 seconds, and 72°c for 5 seconds. The PCR product of the first tube was directly sequenced, whereas the PCR product of the second tube was submitted to a second step in order to improve the sensitivity of the mutation detection. Briefly, BsmaI restriction enzyme was added to this two-steps sample in order to digest the wild-type form of *c-KIT*. The digested PCR product (thus enriched either in D816V or other D816X mutated sequences) was amplified through a nested PCR using the 2365S and 2600AS primers. In addition, nested reaction was performed in the presence of a PNA816 oligonucleotide specific for the wild-type sequence to block amplification of any residual WT sequence.

The two PCR products were then purified using the Illustra ExoProStar kit (GE Healthcare Life Sciences) and were sequenced using the BigDye Terminator v1.1 kit (Applied Biosystems) either with the U2 and SeqL primers for the one step PCR amplification or the 2365S and 2600AS primers for the two steps nested PCR products. Then, the sequencing products were purified using the Sephadex G-50 (GE Healthcare) and a Multiscreen HTS plate (Merck Millipore) and run on an ABI Prism 3730 Genetic Analyzer (Applied Biosystems). The sequences were analyzed using the SeqScape software (Applied Biosystems) by comparison with the NM_000222.2 GeneBank reference sequence (http://www.ncb.nlm.nih.gov).

The whole *c-KIT* coding region corresponding to exons 8 to 13 was sequenced from a PCR amplification using the 1197S and 2029AS primers for 40 cycles at 98°c for 5 seconds, 60°c for 5 seconds, and 72°c for 10 seconds. Then, direct sequencing reactions were performed using the 1197S, 1678AS, 1595S and 2029AS primers.

Table 1. Primers used for *c-KIT* mutation screening

| Primer | Nucleotide Sequence (5’-3’) | Position (NM_000222.2) |
| --- | --- | --- |
| U2 | GGATGACGAGTTGGCCCTAGA | 2274 to 2294 |
| L1 | GTAGAACTTAGAATCGACCGGCA | 2640 to 2618 |
| SeqL | TCGACCGGCATTCCAGGATAGGG | 2627 to 2605 |
| 2365S | ATTCACAGAGACTTGGCAGC | 2365 to 2384 |
| 2600AS | CTTCCTAAAGAGAASAGCTCC | 2600 to 2580 |
| 1197S | CCTAGTGTCCAATTCTGACG | 1176 to 1195 |
| 1678AS | CCTTCCACTGTACTTCAT | 1675 to 1658 |
| 1595S | TGCTGATTGGTTTCGTAATCG | 1574 to 1594 |
| 2029AS | CACCATAGCAACAATATTCTG | 2029 to 2009 |
| PNA816 | TCTAGCCAGAG7^m^5^m^8^m^CATCAAGAATGATT | 2436 to 2461 |

Modifications PNA816 :

7^m^ : C6-carboxylinker 178.12

5^m^ : PNA A 275.4

8^m^ : PNA amine C6 117.2

To determine the sensitivity of the technique, we performed an experiment mixing a HMC1.2 cell line (carrying the D816V mutation) diluted in a *c-KIT* WT expressing cell line (TF1 cells), and the threshold of the two-steps nested PCR amplification procedure was 0.01% for the D816V mutation detection. In order to be in a more physiological context we used the NB4 non-*c-KIT* expressing cell line as dilution cell line and the sensitivity was improved to 0.001%.

# Supplemental Methods 2

### *Data collection*

All the patients in France were enrolled in a prospective study sponsored by AFIRMM. The AFIRMM study was approved by the local investigational review board (Comité de Protection des Personnes Ile-de-France, Pitié-Salpétrière, France; reference: 93-00) and was carried out in compliance with the principles of the Declaration of Helsinki. Written informed consent was obtained from the patients for the publication of data included in this article. All patients from Belgium had been diagnosed at Antwerp University Hospital (Antwerp, Belgium). The collection and storage of personal medical data in the CEREMAST database was authorized by the French National Data Protection Commission (reference CNIL 1445939).

All the French university hospitals in the CEREMAST network were then asked to confirm their data and to add any relevant information that had not been initially collected. In a second phase, three expert centers for mastocytosis in Belgium were invited to participate in the study. Data on the following variables were collected for each patient: date of symptom onset, date of diagnosis, relevant personal and family medical history, clinical features at diagnosis, laboratory variables at diagnosis, BST level, *KIT 816* mutation status (RT-PCR (see Supplemental data 3 for details of the procedure and sensitivity) or droplet digital PCR), *TPSAB1* copy number variation (measured by ddPCR as described by Lyons et al^1^), pathological assessment of a BM biopsy, BM smear examination, BM MC phenotype, bone densitometry data at diagnosis, treatment strategy, and follow-up. BM MC phenotype was not systematically performed in pathological assessment of a BM biopsy with no atypical MC infiltration. In these cases, the result is considered as not available. Anaphylaxis was defined according to the consensus criteria^2,3^, as were osteopenia and osteoporosis^4,5^.

*References*

1. Lyons JJ, Yu X, Hughes JD, Le QT, Jamil A, Bai Y, et al. Elevated basal serum tryptase identifies a multisystem disorder associated with increased TPSAB1 copy number. Nat Genet. 2016 Dec;48(12):1564–9.

2. Sampson HA, Muñoz-Furlong A, Bock SA, Schmitt C, Bass R, Chowdhury BA, et al. Symposium on the Definition and Management of Anaphylaxis: Summary report. Journal of Allergy and Clinical Immunology. 2005 Mar 1;115(3):584–91.

3. Sampson HA, Muñoz-Furlong A, Campbell RL, Adkinson NF, Bock SA, Branum A, et al. Second symposium on the definition and management of anaphylaxis: Summary report—Second National Institute of Allergy and Infectious Disease/Food Allergy and Anaphylaxis Network symposium. Journal of Allergy and Clinical Immunology. 2006 Feb 1;117(2):391–7.

4. Kanis JA, Kanis JA. Assessment of fracture risk and its application to screening for postmenopausal osteoporosis: Synopsis of a WHO report. Osteoporosis Int. 1994 Nov 1;4(6):368–81.

5. NIH Consensus Development Panel on Osteoporosis Prevention, Diagnosis, and Therapy. Osteoporosis Prevention, Diagnosis, and Therapy. JAMA: The Journal of the American Medical Association. 2001 Feb 14;285(6):785–95.

# Supplemental Table 1

| Patients | bST (ng/mL) | CD2+ expression | CD25+ expression | ≥25% of atypical MCs | *KIT* mutation | RT or ddPCR | Number of minor criteria | BM findings |
| --- | --- | --- | --- | --- | --- | --- | --- | --- |
| 1 | 4.1 | 1 | 1 | 0 | 0 | RT-PCR | 1 | Absence of major criteria |
| 2 | 3.6 | 1 | 1 | 0 | 0 | RT-PCR | 1 | Absence of major criteria |
| 3 | 6 | 1 | 1 | 1 | 0 | RT-PCR | 2 | Absence of major criteria |
| 4 | 10 | 1 | 1 | 0 | 0 | RT-PCR | 1 | Absence of major criteria |
| 5 | 18 | 1 | 1 | 1 | 0 | RT-PCR | 2 | Absence of major criteria |
| 6 | 7.6 | 1 | 1 | 0 | D816V | RT-PCR | 2 | Absence of major criteria |
| 7 | 25.4 | 0 | 0 | 0 | D816V | RT-PCR | 2 | Absence of major criteria |
| 8 | 7.3 | 0 | 0 | 0 | D816V | RT-PCR | 1 | Absence of major criteria |
| 9 | 9.2 | 0 | 0 | 0 | D816V | RT-PCR | 1 | Absence of major criteria |
| 10 | 14.2 | NA | NA | 0 | D816V | RT-PCR | 1 or 2 | Absence of major criteria |
| 11 | 15 | 0 | 0 | 0 | D816V | RT-PCR | 1 | Absence of major criteria |
| 12 | 30 | 0 | 0 | 0 | D816V | RT-PCR | 2 | Absence of major criteria |
| 13 | 16.9 | 0 | 0 | 0 | D816V | RT-PCR | 1 | Absence of major criteria |
| 14 | 18.3 | 1 | 1 | 0 | D816V | RT-PCR | 2 | Absence of major criteria |
| 15 | NA | 0 | 1 | 0 | 0 | RT-PCR | 1 or 2 | Absence of major criteria |
| 16 | NA | 1 | 1 | 0 | 0 | RT-PCR | 1 or 2 | Absence of major criteria |
| 17 | 21.2 | 1 | 1 | 0 | 0 | RT-PCR | 2 | Absence of major criteria |
| 18 | 15 | 1 | 1 | 0 | D816V | RT-PCR | 2 | Absence of major criteria |
| 19 | 5 | 0 | 1 | 0 | 0 | RT-PCR | 1 | Absence of major criteria |
| 20 | 16.4 | 1 | 1 | 0 | D816V | RT-PCR | 2 | Absence of major criteria |
| 21 | 13.1 | 1 | 1 | 0 | 0 | ddPCR | 1 | Absence of major criteria |
| 22 | 3.6 | 1 | 1 | 0 | 0 | RT-PCR | 1 | Absence of major criteria |
| 23 | 12.5 | 0 | 0 | 0 | D816V | ddPCR | 1 | Absence of major criteria |
| 24 | 14.9 | 0 | 1 | 0 | 0 | ddPCR | 1 | Absence of major criteria |
| 25 | 20 | 0 | 0 | 0 | D816V | ddPCR | 2 | Absence of major criteria |
| 26 | 17 | 0 | 0 | 0 | D816V | RT-PCR | 1 | Absence of major criteria |
| 27 | 25.4 | 1 | 1 | 0 | 0 | ddPCR | 2 | Absence of major criteria |
| 28 | 8.3 | 0 | 0 | 0 | D816V | ddPCR | 1 | Absence of major criteria |
| 29 | 29.5 | 1 | 1 | 0 | 0 | RT-PCR | 2 | Absence of major criteria |
| 30 | 4.3 | 0 | 0 | 0 | D816V | ddPCR | 1 | Absence of major criteria |
| 31 | 5.7 | 1 | 1 | 0 | 0 | ddPCR | 1 | Absence of major criteria |
| 32 | 13 | NA | NA | 0 | D816V | RT-PCR | 1 or 2 | Absence of major criteria |
| 33 | 7.4 | 0 | 0 | 0 | D816V | ddPCR | 1 | Absence of major criteria |
| 34 | 5.5 | 0 | 1 | 0 | D816V | ddPCR | 2 | Absence of major criteria |
| 35 | 27 | 0 | 1 | 0 | 0 | ddPCR | 2 | Absence of major criteria |
| 36 | 31 | 1 | 1 | 0 | 0 | ddPCR | 2 | Absence of major criteria |
| 37 | 5.8 | 1 | 1 | 1 | 0 | ddPCR | 2 | Absence of major criteria |
| 38 | 7.8 | 1 | 1 | 0 | D816V | ddPCR | 2 | Absence of major criteria |
| 39 | 5.8 | 0 | 1 | 0 | 0 | ddPCR | 1 | Absence of major criteria |
| 40 | 5.8 | 1 | 1 | 0 | D816V | ddPCR | 2 | Absence of major criteria |
| 41 | 66.6 | 1 | 1 | 0 | 0 | ddPCR | 2 | Absence of major criteria |
| 42 | 15.4 | 0 | 1 | NA | D816V | ddPCR | 2 | Absence of major criteria |
| 43 | 6.8 | 0 | 1 | 0 | D816V | ddPCR | 2 | Absence of major criteria |
| 44 | 17 | 0 | 1 | 0 | 0 | RT-PCR | 1 | Absence of major criteria |
| 45 | 35.3 | 0 | 0 | 0 | D816V | RT-PCR | 2 | Absence of major criteria |
| 46 | 10.7 | 1 | 1 | 0 | D816V | ddPCR | 2 | Absence of major criteria |
| 47 | 8.4 | 0 | 0 | 0 | D816V | ddPCR | 1 | Absence of major criteria |
| 48 | NA | 1 | 1 | 0 | 0 | RT-PCR | 1 or 2 | Absence of major criteria |
| 49 | 7.5 | 0 | 0 | 1 | D816V | RT-PCR | 2 | Absence of major criteria |
| 50 | 13.9 | 0 | 0 | 0 | D816V | ddPCR | 1 | Absence of major criteria |
| 51 | 8.2 | 0 | 1 | 0 | D816V | ddPCR | 2 | Absence of major criteria |

Supplemental table 1 legend: BST: basal serum tryptase. MC: Mast cell. ddPCR: Droplet Digital Polymerase Chain Reaction. RT-PCR: Reverse Transcription Polymerase Chain Reaction BM: bone marrow. NA: not available.

# Supplemental Table 2

|  | Symptoms of MCA | MCAS criteria |  |  |  |
| --- | --- | --- | --- | --- | --- |
| Patients |  | Increased tryptase level from bST +20% +2ng/mL | Response MC-stabilizing agents | Number of organs involved | Consensus criteria for MCAS fullfiled |
| 1 | other | NA | NA | 3 | NA |
| 2 | other | NA | NA | 2 | NA |
| 3 | other | NA | NA | 2 | NA |
| 4 | other | NA | 1 | 3 | NA |
| 5 | other | NA | 1 | 3 | NA |
| 6 | HVA with mild other | NA | 1 | 2 | NA |
| 7 | other | NA | 1 | 1 | 0 |
| 8 | HVA with mild other | NA | NA | 1 | 0 |
| 9 | other | NA | NA | 2 | 0 |
| 10 | other | NA | NA | 2 | NA |
| 11 | other | NA | NA | 1 | 0 |
| 12 | other | NA | NA | 2 | NA |
| 13 | HVA | NA | 1 | 1 | 0 |
| 14 | other | NA | 0 | 3 | 0 |
| 15 | other | NA | 1 | 3 | NA |
| 16 | other | NA | 0 | 4 | 0 |
| 17 | HVA | NA | NA | 1 | 0 |
| 18 | HVA | NA | NA | 1 | 0 |
| 19 | other | NA | NA | 3 | NA |
| 20 | other | NA | 1 | 3 | NA |
| 21 | IA | 1 | 1 | 1 | 0 |
| 22 | other | NA | 1 | 3 | NA |
| 23 | other | NA | NA | 1 | 0 |
| 24 | other | NA | 1 | 2 | NA |
| 25 | drug allergy | 1 | NA | 1 | 0 |
| 26 | IA | NA | NA | 1 | 0 |
| 27 | drug allergy | 1 | NA | 1 | 0 |
| 28 | HVA | 1 | NA | 1 | 0 |
| 29 | drug allergy | NA | 1 | 1 | 0 |
| 30 | HVA | NA | NA | 1 | 0 |
| 31 | HVA | NA | NA | 1 | 0 |
| 32 | other | NA | 1 | 1 | 0 |
| 33 | HVA | NA | NA | 1 | 0 |
| 34 | IA | NA | NA | 1 | 0 |
| 35 | IA | 1 | 1 | 1 | 0 |
| 36 | other | NA | NA | 3 | NA |
| 37 | HVA | NA | NA | 1 | 0 |
| 38 | HVA | 1 | NA | 1 | 0 |
| 39 | HVA with mild other | NA | NA | 2 | NA |
| 40 | HVA with mild other | NA | NA | 2 | NA |
| 41 | IA | NA | NA | 1 | 0 |
| 42 | HVA | NA | NA | 1 | 0 |
| 43 | HVA with mild other | NA | NA | 2 | NA |
| 44 | other | NA | NA | 3 | NA |
| 45 | HVA | NA | 1 | 1 | 0 |
| 46 | HVA | 1 | NA | 1 | 0 |
| 47 | HVA | 1 | NA | 1 | 0 |
| 48 | other | NA | NA | 2 | NA |
| 49 | other | NA | 1 | 3 | NA |
| 50 | HVA | NA | NA | 1 | 0 |
| 51 | HVA with mild other | NA | 1 | 1 | 0 |

Supplemental table 2 legend: MCA: mast cell activation. MCAS: mast cell activation syndrome. BST: basal serum tryptase. NA: not available. HVA: Hymenoptera venom allergy, other: non anaphylactic cutaneous involvement, gastrointestinal involvement, musculoskeletal involvement, neuropsychiatric involvement, flushes and malaises.

# Supplemental Table 3

Patient characteristics according to the country of diagnosis

| **General characteristics** | **Belgium** N=23 | | **France** N=28 | **P-value** | | |
| --- | --- | --- | --- | --- | --- | --- |
| Age at diagnosis, y, median [IQR] | 65 [52 – 70] | | 57 [45 – 66] | ns | | |
| Age of first symptoms, y, median [IQR] | 60 [50 – 64] | | 45 [40 – 55] | 0.004 | | |
| Age at diagnosis, y, median [IQR] | 61 [51 – 67] | | 52 [44 – 61] | 0.048 | | |
| Time between symptom onset and diagnosis, y, median [IQR] | 0 [0 – 2] | | 4 [1 – 9] | 0.005 | | |
| Sex ratio, M/F | 1.3 | | 0.75 | ns | | |
| **Medical history** | **n/N (%)** | | **n/N (%)** | **P-value** | | |
| Malignant tumor | 2/22 (9.1) | | 3/27 (11) | ns | | |
| Cardiovascular disease | 1/22 (4.5) | | 5/23 (22) | ns | | |
| Autoimmune disease | 0/22 (0) | | 1/28 (3.6) | ns | | |
| **Diagnostic criteria** |  | |  |  | | |
| Aberrant CD2 expression | 7/23 (30) | | 14/26 (54) | ns | | |
| Aberrant CD25 expression | 14/23 (61) | | 17/26 (65) | ns | | |
| *KIT* mutation status |  | |  | ns | | |
| D816V | 14/23 (61) | | 15/28 (54) |  | | |
| Wild-type *KIT* | 9/23 (39) | | 13/28 (46) |  | | |
| **Symptoms** |  | |  |  | | |
| Anaphylaxis | 20/23 (87) | 20/28 (71) | | | ns |  |
| Cutaneous (pruritus, excessive sweating, dermographism, urticaria) | 1/22 (4.5) | 16/28 (57) | | | <0.001 |  |
| Gastrointestinal | 6/23 (26) | 16/28 (57) | | | 0.026 |  |
| Flushing | 1/23 (4.3) | 12/28 (43) | | | 0.002 |  |
| Neuropsychiatric | 1/23 (4.3) | 11/24 (46) | | | 0.001 |  |
| Musculoskeletal | 1/23 (4.3) | 6/23 (26) | | | ns |  |
| Malaises | 0/23 (0) | 5/28 (18) | | | ns |  |
| Asthenia | 1/23 (4.3) | 7/22 (32) | | | 0.022 |  |
| Splenomegaly | 1/14 (7.1) | 0/28 (0) | | | ns |  |
| Osteoporosis | 3/3 (100) | 7/19 (37) | | | ns |  |

# Supplemental Table 4

| **Femoral and lumbar T-scores in patients with MMCS and patients with ISM**  **or BMM (controls)** | | | | | | |
| --- | --- | --- | --- | --- | --- | --- |
| **T-score** | **MMCS** N=21 | **ISM** N=124 | ***P*-value** | **BMM** N=51 | ***P*-value** |  |
| Femoral T-score, SD [IQR] | -1.45 [-2.18 – -1.28] | -1.60 [-2.10 – -1.00] | ns | -1.65 [-2.18 – -1.23] | ns |  |
| Lumbar T-score, SD [IQR] | -2.55 [-2.90 – -2.08] | -2.00 [-2.65 – -1.40] | ns | -1.90 [-2.68 – -1.58] | ns |  |

# Supplemental Table 5

Adjusted analysis (on age, sex, time from symptom onset to diagnosis) for patient characterics and clinical presentation (MMCS vs. ISM)

| **Medical history** | **N** | | **exp(Beta)** | **95% CI** | | | **p-value** | |  |
| --- | --- | --- | --- | --- | --- | --- | --- | --- | --- |
| Malignant tumor | 339 | | 1.01 | 0.90 to 1.14 | | | ns | |  |
| Cardiovascular disease | 319 | | 0.94 | 0.84 to 1.05 | | | ns | |  |
| Autoimmune disease | 335 | | 0.93 | 0.80 to 1.09 | | | ns | |  |
| **Diagnostic criteria** |  | |  |  | | |  | |  |
| Aberrant CD2 expression | 204 | | 0.63 | 0.56 to 0.71 | | | <0.001 | |  |
| Aberrant CD25 expression | 207 | | 0.65 | 0.56 to 0.76 | | | <0.001 | |  |
| *KIT* D816V mutation | 268 | | 0.43 | 0.37 to 0.49 | | | <0.001 | |  |
| **Symptoms** |  | |  |  | | |  |  |  |
| Anaphylaxis | 340 | 1.25 | | | 1.16 to 1.34 | <0.001 | | | |
| Cutaneous (pruritus, excessive sweating, dermographism, urticaria) | 367 | 0.60 | | | 0.55 to 0.65 | <0.001 | | | |
| Gastrointestinal | 358 | 0.88 | | | 0.82 to 0.95 | <0.001 | | | |
| Flushing | 356 | 0.81 | | | 0.76 to 0.87 | <0.001 | | | |
| Neuropsychiatric | 353 | 0.84 | | | 0.79 to 0.90 | <0.001 | | | |
| Musculoskeletal | 226 | 0.63 | | | 0.58 to 0.69 | <0.001 | | | |
| Malaises | 216 | 0.78 | | | 0.70 to 0.88 | <0.001 | | | |
| Asthenia | 235 | 0.66 | | | 0.61 to 0.72 | <0.001 | | | |
| Splenomegaly | 216 | 0.87 | | | 0.72 to 1.06 | ns | | | |
| Osteoporosis | 112 | 1.12 | | | 0.96 to 1.31 | ns | | | |

# Supplemental Table 6

Adjusted analysis (on age, sex, time from symptom onset to diagnosis) for patient characterics and clinical presentation (MMCS vs. BMM)

| **Medical history** | **N** | | **exp(Beta)** | **95% CI** | | | **p-value** | |  |
| --- | --- | --- | --- | --- | --- | --- | --- | --- | --- |
| Malignant tumor | 92 | | 1.07 | 0.74 to 1.55 | | | ns | |  |
| Cardiovascular disease | 87 | | 0.89 | 0.66 to 1.18 | | | ns | |  |
| Autoimmune disease | 91 | | 0.71 | 0.45 to 1.13 | | | ns | |  |
| **Diagnostic criteria** |  | |  |  | | |  | |  |
| Aberrant CD2 expression | 90 | | 0.54 | 0.44 to 0.65 | | | <0.001 | |  |
| Aberrant CD25 expression | 91 | | 0.57 | 0.45 to 0.73 | | | <0.001 | |  |
| *KIT* D816V mutation | 0.54 | | 0.44 to 0.67 | <0.001 | | | ns | |  |
| **Symptoms** |  | |  |  | | |  |  |  |
| Anaphylaxis | 95 | 1.06 | | | 0.82 to 1.37 | ns | | | |
| Cutaneous (pruritus, excessive sweating, dermographism, urticaria) | 94 | 1.13 | | | 0.90 to 1.42 | ns | | | |
| Gastrointestinal | 95 | 1.00 | | | 0.80 to 1.23 | ns | | | |
| Flushing | 95 | 0.65 | | | 0.54 to 0.78 | <0.001 | | | |
| Neuropsychiatric | 91 | 0.75 | | | 0.61 to 0.94 | 0.011 | | | |
| Musculoskeletal | 90 | 0.70 | | | 0.56 to 0.87 | 0.001 | | | |
| Malaises | 94 | 0.63 | | | 0.49 to 0.79 | <0.001 | | | |
| Asthenia | 89 | 0.81 | | | 0.64 to 1.04 | ns | | | |
| Splenomegaly | 86 | 0.91 | | | 0.50 to 1.65 | ns | | | |
| Osteoporosis | 44 | 1.16 | | | 0.83 to 1.62 | ns | | | |

# Supplemental Table 7A

Multivariate analysis for characteristics of patients (MMCS vs. ISM)

| **Characteristics** | OR | 95% CI | p-value |
| --- | --- | --- | --- |
| Time between symptom onset and diagnosis, y, median [IQR] | 0.91 | 0.83 to 0.98 | 0.033 |
| Aberrant CD2 expression | 0.08 | 0.02 to 0.25 | <0.001 |
| *KIT* D816V mutation | 0.06 | 0.02 to 0.19 | <0.001 |

# Supplemental Table 7B

Multivariate analysis for clinical presentation (MMCS vs. ISM)

| **Symptoms** | OR | 95% CI | p-value |
| --- | --- | --- | --- |
| Musculoskeletal | 0.09 | 0.02 to 0.34 | <0.001 |
| Malaises | 0.09 | 0.00 to 0.61 | 0.036 |
| Asthenia | 0.09 | 0.02 to 0.34 | <0.001 |

# Supplemental Table 8A

Multivariate analysis for characteristics of patients (MMCS vs. BMM)

| **Characteristics** | OR | 95% CI | p-value |
| --- | --- | --- | --- |
| Aberrant CD2 expression | 0.01 | 0.00 to 0.12 | 0.003 |
| *KIT* D816V mutation | 0.03 | 0.01 to 0.15 | <0.001 |

# Supplemental Table 8B

Multivariate analysis for clinical presentation (MMCS vs. BMM)

| **Symptoms** | OR | 95% CI | p-value |
| --- | --- | --- | --- |
| Flushing | 0.16 | 0.04 to 0.56 | 0.007 |
| Musculoskeletal | 0.18 | 0.03 to 0.91 | 0.049 |
| Malaises | 0.04 | 0.00 to 0.36 | 0.018 |

# Supplemental Table 9

Treatments received: patients with MMCS versus patients with ISM or BMM (controls)

|  | **MMCS** N=51 | **ISM** N=432 | ***P*-value** | **BMM** N=51 | ***P*-value** |
| --- | --- | --- | --- | --- | --- |
| **Treatments** | **n/N (%)** | **n/N (%)** |  | **n/N (%)** |  |
| H1 antihistamines | 25/50 (51) | 339/432 (78) | <0.001 | 34 (67) | ns |
| H2 antihistamines | 18/50 (36) | 212/432 (49) | ns | 13 (25) | ns |
| Sodium cromoglycate | 7/50 (14) | 102/432 (24) | ns | 6 (12) | ns |
| Montelukast | 12/50 (24) | 127/432 (29) | ns | 13 (25) | ns |
| Steroids | 3/50 (6) | 44/432 (10) | ns | 6 (12) | ns |
| Rapamycin | 0/50 (0) | 6/432 (1) | ns | 1 (2) | ns |
| α-Interferon | 0/50 (0) | 39/432 (9) | 0.024 | 1 (2) | ns |
| Imatinib | 0/50 (0) | 19/432 (4) | ns | 0/50 (0) | ns |
| Omalizumab | 6/50 (12) | 9/177 (5) | ns | 8 (17) | ns |

# Supplemental Figure 1

Final diagnosis for patients in the cohort

1. Clinical presentation (proportions of osteoporosis/osteopenia among the three groups are represented within the bars)

1. Allergy distribution
