# Supplementary material for: Characterization of patients with clonal mast cells in the bone marrow with clinical significance not otherwise specified
Source: eClinicalMedicine. 2025 Jan 10;80:103043. doi: 10.1016/j.eclinm.2024.103043 (PMC11773267; doi:10.1016/j.eclinm.2024.103043)
Supplement: CEREMAST study group [file mmc2.docx]

**CEREMAST study group :**

Thomas Ballul MD^1^, Vito Sabato MD, PhD^2^, Cristina Bulai Livideanu MD^3^, Antoine Neuraz MD, PhD^4^, Julie Agopian MSc^5^, Fabienne Brenet PhD^5^, Patrice Dubreuil PhD^1,5^, Didier G Ebo MD, PhD^2^, Michiel Beyens MD, PhD^2^ Richard Lemal MD, PhD^7,8^, Olivier Tournilhac MD, PhD^8^, Louis Terriou MD^9^, David Launay MD, PhD^9,10^, Laurence Bouillet MD, PhD^11^, Catharina Chatain MD^12^, Clément Gourguechon MD^13^, Gandhi Damaj MD, PhD^14^, Stéphane Durupt MD^15^, Celine Greco MD, PhD^16^, Laurent Frenzel MD, PhD^1^, Christine Bodemer-Skandalis MD, PhD^17^, Laura Polivka MD, PhD^17^, Marine Madrange MSc^1^, Cécile Meni MD^1^, Hassiba Bouktit MSc^1^, Anne Florence Bellais^1^, Jean-Marc Durand MD, PhD^18^, Marie Gousseff MD^19^, Edwige Le Mouel MD^20^, Mohamed Hamidou MD,PhD^21^, Antoine Neel MD, PhD^21^, Dana Ranta MD^22^, Mathilde Niault MD^23^, Aurélie Schiffmann MD^24^, Stéphane Barete MD, PhD^25^, Michel Arock MD, PhD^26^, Danielle Canioni MD^27^, Thierry Jo Molina MD, PhD^27^, Julie Bruneau MD, PhD^27^, Mélanie Vaes MD^28^, Violaine Havelange MD, PhD^29^, Hassan Faour PhD^30^, Nicolas Garcelon PhD^30^, Rose-Marie Javier MD^31^, Fabien Pelletier MD, PhD^32^, Florence Castelain MD^32^, Denis Vincent MD, PhD^33^, Frédérique Retornaz MD^34^, Quentin Cabrera MD^35^, Patricia Zunic MD, PhD^35^, Philippe Guilpain MD, PhD^24^, Marie Pierre Gourin MD^36^, Ewa Wierzbicka–Hainaut MD, MSc^37^, Jean François Viallard MD, PhD^38^, Christian Lavigne MD, PhD^39^, Cyrille Hoarau MD, PhD^40^, Ludovic Lhermitte MD, PhD^41^, Maël Heiblig MD^42^, Roland Jaussaud MD, PhD^43^, Peter Valent MD, PhD^44,45^, Olivier Hermine MD, PhD^1°*^, Olivier Lortholary MD, PhD^1°*^, Julien Rossignol MD, PhD^1°^

1. French Reference Center for Mastocytosis (CEREMAST), Paris Cité University, Necker –Enfants Malades University Hospital, APHP, Paris, France
2. Department of Immunology Allergology and Rheumatology University of Antwerp and Antwerp University Hospital, Antwerp, Belgium
3. French Reference Center for Mastocytosis (CEREMAST), Department of Dermatology, Hôpital Larrey, CHU Toulouse, Toulouse, France
4. Department of Bioinformatics, Necker Branch, Necker-Enfants Malades Hospital (AP-HP), Paris-Centre University, Imagine Institute, INSERM U1163, Paris, France

Centre de Recherche en Cancérologie de Marseille, INSERM U1068, Marseille, France.

Association Française pour les Initiatives de Recherche sur le Mastocyte et les Mastocytoses (AFIRMM), Marseille, France

1. Histocompatibility Laboratory, EA 7453—Université Clermont Auvergne, CHU de Clermont-Ferrand, Clermont-Ferrand, France
2. Adult Clinical Hematology, CHU Clermont-Ferrand, INSERM CIC501, EA 7453 -Université Clermont Auvergne, Clermont-Ferrand, France
3. Department of Internal Medicine and Clinical Immunology, Hôpital Claude Huriez, CHRU Lille, Lille, France
4. Université Lille, INSERM U995 LIRIC, CHU Lille, and Referral Center for Rare Systemic Autoimmune Diseases North and North-West of France, Lille, France
5. Clinical Immunology/Internal Medicine Department, National Reference Center for Angioedema, Grenoble University Hospital, Grenoble, France
6. Dermatology, Allergology and Photobiology Department, Grenoble University Hospital, Grenoble, France
7. Department of Internal Medicine, Amiens University Hospital, France
8. Haematology Institute, Normandy University School of Medicine, Caen, France.
9. Department of Internal Medicine, Adult Cystic Fibrosis Care Center, Hospices Civils de Lyon, Lyon, France
10. French Reference Center for Mastocytosis (CEREMAST), Department of Pain and Palliative Care Unit, Hôpital Universitaire Necker Enfants Malades, Assistance Publique Hôpitaux de Paris, Paris, France
11. Department of Paediatric Dermatology, French Reference Centre for Pediatric Mastocytosis (CEREMAST) and Rare Skin Disorders (MAGEC), Necker-Enfants Malades Hospital , Paris Centre University Assistance Publique Hôpitaux de Paris, Paris, France
12. Internal Medicine Department, Aix-Marseille University, Hopital Timone Rue Saint Pierre, Marseille, France
13. Department of Internal Medicine, Centre Hospitalier Bretagne Atlantique, Vannes, France
14. Department of Internal Medicine and Clinical Immunology, Rennes University Hospital, Rennes, France
15. Department of Internal Medicine, Hôtel-Dieu University Hospital, Nantes, France
16. Department of Haematology, Nancy University Hospital, Nancy, France
17. Hematology Department, Lorient Hospital, Lorient, France
18. Department of Internal Medicine-Multi-organ Diseases, Saint-Eloi University Hospital, University of Montpellier, Montpellier, France
19. French Reference Center for Mastocytosis (CEREMAST), Dermatology Department, Pitié-Salpêtrière Hospital, AP-HP, Paris, France
20. French Reference Center for Mastocytosis (CEREMAST), Laboratory of Hematology, Pitié-Salpêtrière Hospital, AP-HP, Paris, France.
21. Department of Pathology, Université Paris Cité, Necker-Enfants Malades Hospital, APHP, Paris, France
22. Department of Hematology, Université Libre de Bruxelles, Hôpital Erasme, Brussels, Belgium; Department of Hematology, Université Libre de Bruxelles, CHU Tivoli, La Louvière, Belgium
23. Department of Hematology, Cliniques Universitaires St. Luc, Brussels, Belgium
24. Université de Paris, Imagine Institute, Data Science Platform, INSERM UMR 1163, F-75015, Paris, France
25. Department of Rheumatology, Strasbourg University Hospital, Strasbourg, France.
26. Department of Dermatology, Besancon University Hospital, Besancon, France.
27. Department of Pneumology and Internal Medicine, Caremeau University Hospital, Nimes, France.
28. Department of Internal Medicine, European Hospital, Marseille, France.
29. Department of Haematology, Sud Reunion University Hospital, Saint Pierre, La Réunion, France.
30. Department of Hematology, CHU Dupuytren, Limoges, France.
31. Department of Dermatology, CHU de Poitiers, Poitiers, France.
32. Department of Internal Medicine and Infectious Diseases, Hôpital Haut-Lévêque, CHRU Bordeaux; University of Bordeaux, BORDEAUX, France.
33. Department of Internal Medicine and Clinical Immunology, University Hospital, Angers, France.
34. Transversal Unit of Allergology and Clinical Immunology, Medicine Department, Tours Regional University Hospital, Tours University, France.
35. French Reference Center for Mastocytosis (CEREMAST), Laboratory of onco-hematology, Necker Children's Hospital, APHP, Paris, France
36. Department of Hematology, Lyon-Sud Hospital, Hospices Civils de Lyon, Pierre-Bénite, France.
37. Department of Internal Medicine and Clinical Immunology, Vandoeuvre-lès-Nancy, France.
38. Department of Internal Medicine I, Division of Hematology and Hemostaseology, Medical University of Vienna, Austria.
39. Ludwig Boltzmann Institute for Hematology and Oncology, Medical University of Vienna, Austria.
